# *N*-acetylcysteine Facilitates Self-Imposed Abstinence After Escalation of Cocaine Intake

**DOI:** 10.1016/j.biopsych.2015.09.019

**Published:** 2016-08-01

**Authors:** Eric Ducret, Mickaël Puaud, Jérôme Lacoste, Aude Belin-Rauscent, Maxime Fouyssac, Emilie Dugast, Jennifer E. Murray, Barry J. Everitt, Jean-Luc Houeto, David Belin

**Affiliations:** a French Institute of Health and Medical Research, Avenir Team Psychobiology of Compulsive Disorders, Université de Poitiers, Poitiers, France; b Department of Pharmacology, University of Cambridge, Cambridge, United Kingdom; c Service de Psychiatrie et Addictologie, Centre Hospitalier Universitaire de Fort-de-France, Martinique, France; d Department of Psychology, University of Cambridge, Cambridge, United Kingdom; e Service de Neurologie de l’Hôpital de Poitiers and Center for Clinical Investigation-French Institute of Health and Medical Research, Poitiers, France

**Keywords:** Addiction, Cocaine, Compulsivity, Motivation, *N*-acetylcysteine, Zif268

## Abstract

**Background:**

*N*-acetylcysteine (NAC) has been suggested to prevent relapse to cocaine seeking. However, the psychological processes underlying its potential therapeutic benefit remain largely unknown.

**Methods:**

We investigated the hallmark features of addiction that were influenced by chronic NAC treatment in rats given extended access to cocaine: escalation, motivation, self-imposed abstinence in the face of punishment, or propensity to relapse. For this, Sprague Dawley rats were given access either to 1 hour (short access) or 6 hours (long access [LgA]) self-administration (SA) sessions until LgA rats displayed a robust escalation. Rats then received daily saline or NAC (60 mg/kg, intraperitoneal) treatment and were tested under a progressive ratio and several consecutive sessions in which lever presses were punished by mild electric foot shocks.

**Results:**

NAC increased the sensitivity to punishment in LgA rats only, thereby promoting abstinence. Following the cessation of punishment, NAC-treated LgA rats failed to recover fully their prepunishment cocaine intake levels and resumed cocaine SA at a lower rate than short access and vehicle-treated LgA rats. However, NAC altered neither the escalation of SA nor the motivation for cocaine. At the neurobiological level, NAC reversed cocaine-induced decreases in the glutamate type 1 transporter observed in both the nucleus accumbens and the dorsolateral striatum. NAC also increased the expression of Zif268 in the nucleus accumbens and dorsolateral striatum of LgA rats.

**Conclusions:**

Our results indicate that NAC contributes to the restoration of control over cocaine SA following adverse consequences, an effect associated with plasticity mechanisms in both the ventral and dorsolateral striatum.

There is increasing evidence that cocaine addiction is associated with alterations of glutamate homeostasis within the corticostriatal circuitry (1), the normalization of which by the cysteine prodrug *N*-acetylcysteine (NAC), a substrate for the cysteine/glutamate antiporter, has been suggested to decrease cocaine seeking and promote abstinence in preclinical and clinical studies (2, 3, 4, 5, 6). However, the psychological and neurobiological mechanisms whereby NAC facilitates abstinence are not fully understood. Indeed, in a double-blind placebo-controlled trial (4), NAC treatment resulted neither in abstinence nor in decreases in cocaine intake in addicted individuals actively using the drug. Instead, NAC prevented the return to cocaine use in patients that had already achieved abstinence.

This observation suggests that the normalization to drug naïve control levels by NAC (7) of the decreased glutamate levels observed in cocaine addicts (8, 9) may not be sufficient to promote abstinence and decrease craving (10) but may consolidate the long-lasting effect of the factors that resulted in self-initiated abstinence. Thus, even though preclinical studies have revealed that NAC treatment decreases cue- and drug-induced reinstatement of an extinguished behavioral response for cocaine or prevents the development of escalation of cocaine self-administration (SA) by restoring altered glutamate homeostasis in the nucleus accumbens core (AcbC) (11, 12, 13, 14), the precise potential mechanisms whereby NAC may exert its therapeutic benefits, namely the maintenance of self-initiated abstinence or restored control, have not been established.

Abstinence in humans is often self-initiated following exposure to the negative consequences of drug use in individuals who have lost control over their habitual cocaine intake (15). Therefore, we hypothesized that NAC treatment may help restore control over intake following repeated exposure to punishment in rats displaying escalation of cocaine SA (16) and associated increased motivation for the drug (17).

Thus, we investigated the influence of chronic NAC treatment on the escalation of cocaine intake, increased motivation for the drug, and the propensity to develop and maintain abstinence in the face of punishment, three criteria suggested to define a state of addiction in rats (18). Rats were given either long access sessions (LgA) that drive escalation of cocaine SA or short access (ShA) daily sessions (19). We then assessed motivation for drug in both groups using a progressive ratio (PR) reinforcement schedule (20), followed by punishing cocaine seeking using contingent mild foot shocks (20, 21).

Neurobiologically, in extinction-reinstatement procedures, treatment with NAC has been shown in rats to reverse cocaine-induced glutamatergic adaptations selectively in the AcbC (1), where pharmacogenetically induced astrocytic release of glutamate also decreases cue-induced reinstatement of instrumental responding for cocaine (22). Thus, NAC increases glial expression of the cysteine/glutamate antiporter and glutamate type 1 transporter (GLT1), thereby restoring extracellular levels of glutamate (11, 23) and decreasing excitatory synaptic transmission in the AcbC. However, studies based on acute exposure to cocaine SA followed by extinction may not fully capture the nature of addiction that does not result from an adaptation merely to short-term exposure to the drug under continuous reinforcement (18). Instead, it involves the progressive development of compulsive drug seeking and loss of control over drug intake (24), the neurobiological substrates of which encompass both the ventral striatum and the dorsolateral striatum (DLS) in humans (25, 26) and in preclinical models (27, 28, 29, 30).

Therefore, we hypothesized that the potential influence of NAC treatment over punishment-induced self-abstinence in rats may be related to a normalization of levels of GLT1, the key molecular regulator of astrocytic-dependent glutamate homeostasis in both the AcbC and the DLS, and that the two structures may undergo similar plasticity mechanisms (31, 32) following self-initiated abstinence. Thus, we measured in the striatum of vehicle- and NAC-treated ShA and LgA rats the level of GLT1 and Zif268 proteins, the latter being involved both in instrumental learning-dependent plasticity mechanisms in both the ventral striatum and DLS and also in long-term cocaine-induced neuronal plasticity (31, 32, 33, 34, 35, 36).

## Methods and Materials

### Animals

Sixty male Sprague Dawley rats (Janvier, Saint Berthevin, France) weighing 280–290 g on arrival were housed in pairs under reversed light/dark cycle (lights on at 7:00 am) as described in the Supplement. All experimental procedures complied with the European and French ethical guidelines.

### Solutions

*N*-acetylcysteine was prepared and administered daily as previously described (6). See the Supplement for further details.

### Surgery

Surgical procedures have been described previously (37) and are detailed in the Supplement.

### Apparatus

The 12 operant chambers used in this study have been described previously (37). See the Supplement for more details.

### Procedures

**Figure 1:**
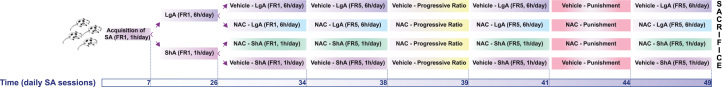
Timeline of the experimental procedure. Following intravenous surgery, rats were allowed one week of recovery before being exposed daily to cocaine self-administration (SA) under a fixed ratio 1 (FR1) reinforcement schedule. After a week, differential access to cocaine was introduced, with half the rats maintained under a 1-hour daily access schedule (short access group [ShA]) and the other half given a daily access to cocaine of 6 hours (long access group [LgA]). Following 19 sessions of differential access, rats were subjected to chronic daily intraperitoneal injections of either *N*-acetylcysteine (NAC) (60 mg/kg) or vehicle until the end of the experiment. After 8 days of treatment onward, the response requirement was increased to a fixed ratio 5 (FR5) reinforcement schedule. Motivation for cocaine was assessed on day 38 with a progressive ratio schedule of reinforcement. After three baseline sessions, rats were exposed to three consecutive punishment sessions during which every fourth and fifth lever press of each instrumental sequence (FR5) delivered a mild foot shock (.3 mA, 1 second). The influence of punishment-induced abstinence on individual propensity to relapse to escalated cocaine self-administration was measured over five sessions following the termination of punishment.

The details of the procedures and the timeline of the experiment are described in Figure 1 and the Supplement.

### Western Blot

The details of the Western blot procedures are described in the Supplement. Each Western blot was run in triplicate on proteins extracted from pooled bilateral punches of the AcbC or anterior DLS. The optical density of each band was normalized by the amount of cyclophilin A present in each well to correct for variations in the quantity of proteins loaded, and the resulting ratio was used to assess protein concentrations. Optical density × area calculation was performed to derive an amount of protein.

### Data and Statistical Analysis

Data are expressed as means ± SEM. The behavioral data were subjected to repeated-measures analyses of variance, while Western blot data were subjected to factorial analyses of variance (see the Supplement for more details). Upon confirmation of main effects, pairwise comparisons were performed using the Newman-Keuls post hoc test.

Statistical significance was claimed at α = .05. For Western blot analyses, because of the heterogeneity of the samples, partial eta-squared values (partial η^2^) are reported as the measure of effect size to support the *p* values (38).

## Results

LgA rats did not differ from ShA rats in their acquisition of cocaine SA but displayed a robust escalation of intake soon after the introduction of extended access, as expected (Supplemental Results and Supplemental Figure S1). Following 19 days of differential access, we measured the influence of chronic NAC treatment on the persistence of loss of control over cocaine intake when more effort was required to obtain the drug, the motivation for the drug, and the propensity to refrain from, and relapse to, drug self-administration. For this, half the animals of each group received daily intraperitoneal injections of either NAC (ShA group: *n* = 10, LgA group: *n* = 9) or vehicle (Veh) (ShA group: *n* = 9, LgA group: *n* = 8) until the end of the experiment (Figure 1).

### NAC Neither Impedes the Progression of Escalation of Cocaine Self-Administration nor Decreases the Motivation for Cocaine

**Figure 2:**
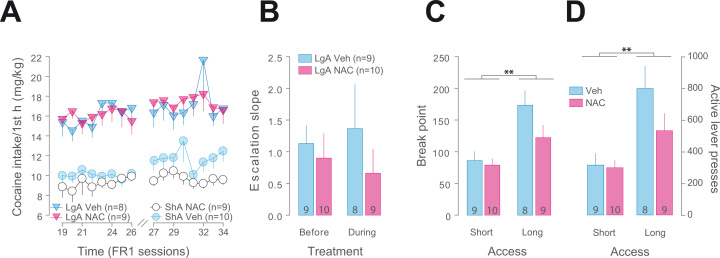
Chronic *N*-acetylcysteine (NAC) treatment alters neither the progression of escalation of cocaine intake nor the motivation for the drug. NAC had no influence on the escalation of cocaine self-administration displayed by long access group (LgA) rats (main effect of access: *F*_1,32_ = 53.662, *p* < .001), as shown by the lack of effect on cocaine intake (main effect of treatment: *F*_1,32_ < 1, and access × treatment × sessions × block _before/during treatment_ interaction: *F*_7,224_ < 1) **(A)** and the escalation slope (treatment × block _before/during treatment_ interaction: *F*_1,15_ < 1) **(B)**. The increased motivation in LgA rats, as revealed by a higher breakpoint under a progressive ratio schedule of reinforcement, compared with short access (ShA) rats (main effect of access: *F*_1,32_ = 14.85, *p* < .01) was not influenced by NAC (main effect of treatment: *F*_1,32_ = 2.94, *p* > .09, and treatment × access interaction: *F*_1,32_ = 1.68, *p* > .2) **(C)**. Similarly, the level of instrumental responding under the progressive ratio schedule, higher in LgA rats than ShA rats (main effect of access: *F*_1,32_ = 14.43, *p* < .0001), was not influenced by NAC (main effect of treatment: *F*_1,32_ = 2.23, *p* > .14, and treatment × access interaction: *F*_1,32_ = 1.72, *p* > .19) **(D)**. Numbers between brackets correspond to the number of animals in each group. Breaks represent the beginning of the treatment. **p < .01. FR1, fixed ratio 1; Veh, vehicle.

NAC treatment had no effect on the progression of the escalation of cocaine intake in LgA rats (Figure 2A). The lack of effect of NAC treatment over the maintenance of escalated cocaine SA (Supplemental Figure S2) was further supported by a similar escalation slope (Figure 2B) in NAC- and Veh-treated LgA rats and thus despite a major difference in intake during the first hour between LgA and ShA rats.

NAC had no effect on the propensity to engage in more effort to obtain cocaine in both ShA and LgA rats when more effort (i.e., training under fixed ratio [FR] 5) was required to self-administer the drug (see the Supplement for further details). When rats were subsequently challenged under a PR schedule of reinforcement (21), NAC treatment had no effect on the motivation for cocaine of ShA and LgA rats, despite a clear enhancement of individual propensity to lever press in the latter (Figure 2C, D). The trend toward a decreased breakpoint in NAC-treated LgA rats did not reach statistical significance (*F*_1,15_ = 2.88, *p* > .11), nor did the trend in the total lever presses emitted during the test (Figure 2D) (*F*_1,15_ = 2.28, *p* > .15); these trends cannot be attributable to differential cocaine exposure before the session or to a differential level of instrumental responding (LgA: *r* = −.25, *R*^*2*^ = .06, *p* > .3 and *r* = −.23, *R*^*2*^ = .05, *p* > .4; ShA: *r* = .22, *R*^*2*^ = .05, *p* > 0.3 and *r* = .19, *R*^*2*^ = .04, *p* > .4 for total cocaine intake and total lever presses up to the PR session, respectively).

### NAC Increases the Sensitivity to Punishment and Promotes Self-Maintained Control Over Cocaine Intake at Relapse

**Figure 3:**
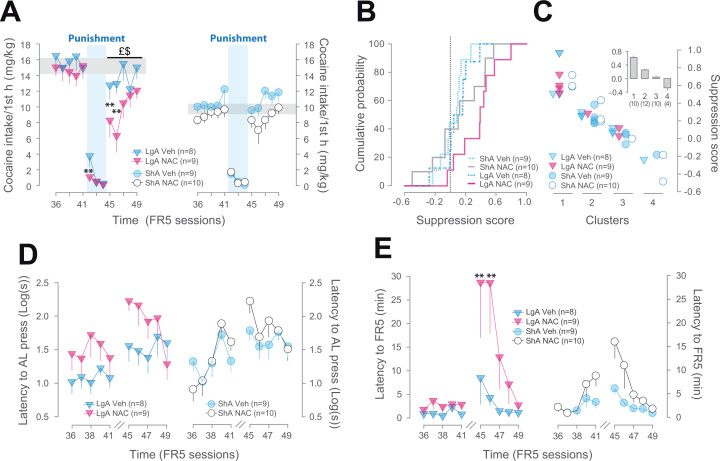
*N*-acetylcysteine (NAC) promotes self-maintained abstinence by increasing the sensitivity to the adverse consequences associated with cocaine intake. Punishment of cocaine self-administration induced a rapid decrease in intake in all groups (main effect of block _FR5/punishment_: *F*_1,32_ = 350.88, *p* < .01). NAC-long access group (LgA) rats decreased their intake to the level of short access group (ShA) rats faster than vehicle (Veh)-LgA rats (treatment × access × sessions interaction: *F*_2,64_ = 3.93, *p* < .05) **(A)**. Following the three punishment sessions (vertical blue bars), NAC-LgA rats recovered prepunishment levels of cocaine intake at a slower rate than all the other groups (effect of block _pre / post punishment_: *F*_1,32_ = 200.85, *p* < .01, treatment × punishment interaction: *F*_1,32_ = 4.22, *p* < .05). A cumulative probability curve **(B)** on the suppression score revealed a rightward shift of the curve for NAC-LgA animals (vertical black dotted line represents a suppression score of 0). At the individual level, a cluster analysis **(C)** identified that the group of rats with the highest suppression score was essentially composed of NAC-LgA rats. Insert **(C)** represents the mean suppression score for each cluster. While NAC treatment did not specifically influence the increased latency to initiate cocaine-seeking behavior following punishment (main effect of punishment _(pre/post block):_*F*_1,32_ = 13.19, *p* < .001, treatment: *F*_1,32_ = 2.76, *p* > .1, and punishment × access × treatment interaction: *F*_1,32_ < 1) **(D)** (data are expressed in log of latency of the first active lever [AL] press), it selectively increased in NAC-LgA rats the time needed to complete the fixed ratio 5 (FR5) instrumental sequence leading to the first cocaine infusion (access × punishment × treatment interaction: *F*_1,32_ = 7.70, *p* < .01) **(E).** Breaks **(D, E)** represent the punished sessions. **Sessions differing from Veh-LgA group, *p* < .01; £, different from prepunishment block, *p* < .05; $, block different from Veh-LgA, *p* ≤ .05. Gray bands represent the average intake during the prepunishment block ± SEM.

Rats progressively developed abstinence in the presence of adverse consequences operationalized by three sessions of contingent foot shock (20, 39) that eventually resulted in a complete cessation of cocaine intake in all experimental groups (Figure 3A). However, the marked differences observed in the development of abstinence were dependent upon both access and treatment conditions. Thus, while ShA rats, regardless of their treatment, stopped self-administering cocaine from the first punishment session, Veh-treated LgA rats displayed resistance to punishment, a behavioral feature of compulsivity (18), in that they maintained a higher level of cocaine intake than all the other experimental groups during the first session (*p*s < .01).

NAC-treated LgA rats, however, displayed a reduction in cocaine intake that was similar to that observed in both Veh-ShA and NAC-ShA rats, thereby differing from Veh-LgA rats (Figure 3A). These differences were dissociable from the effect of punishment on the propensity to initiate the first drug-seeking response from one session to another that dramatically increased over the course of punished sessions regardless of both access and treatment conditions (Supplemental Figure S4A).

Once abstinence was established for 2 days, rats were returned to baseline conditions and resumed cocaine SA. NAC-treated LgA rats did not fully recover their prepunishment level of cocaine intake. Thus, in the first postpunishment session, all ShA and only Veh-treated LgA rats resumed a level of cocaine SA similar to that displayed before the introduction of punishment (*p* > .9). In marked contrast, NAC-LgA rats had significantly reduced their cocaine intake during the 2 days immediately following the shock sessions as compared with cocaine intake during any of the prepunishment sessions (*p* < .01).

To better investigate the potential contribution of baseline differences between LgA and ShA rats to their propensity to resume cocaine self-administration after shock-induced voluntary abstinence, we computed a suppression score (Supplement), which revealed a pronounced rightward shift in the cumulative probability curve for the NAC-LgA group (Figure 3B) as compared with the ShA and Veh-LgA groups. A cluster analysis performed on the suppression ratio identified a group of 10 rats with a postpunishment suppression of cocaine intake of 77 ± 10% (Figure 3C), containing predominantly NAC-LgA rats (*n* = 6). The other clusters represented rats with a suppression of less than 50% that were mostly in the ShA and Veh-LgA groups (*p* < .05), thereby demonstrating that the restored control in NAC-LgA rats cannot be attributable to prepunishment differences.

This NAC-induced reduction of cocaine intake was not attributable to a difference in the latency to initiate the instrumental response, which was increased after the punishment sessions (Figure 3D) independent of treatment and access to cocaine. However, NAC-induced decrease of cocaine self-administration following the cessation of punishment was associated with a marked increase in the time taken by the rats to perform the subsequent four lever presses necessary to complete the FR5 sequence required to obtain the first cocaine infusion (Figure 3E). Thus, the time between the first lever press and the first infusion was significantly increased for NAC-LgA rats during the two sessions following punishment (*p* < .01), whereas no significant alteration of the time of FR5 completion was observed for ShA rats and Veh-LgA animals (*p* > .3). This suggests that if NAC treatment did not influence the initiation of cocaine-seeking behavior in addicted rats—i.e., LgA rats—once this rather automatic behavioral sequence was initiated, NAC did restore control over subsequent instrumental behavior.

### *N*-acetylcysteine Rescues Cocaine-Induced Decreases in GLT1 Protein Levels and Opposes the Influence of Cocaine Exposure Over Zif268 Protein Levels in Both the Ventral Striatum and DLS of LgA Rats

**Figure 4:**
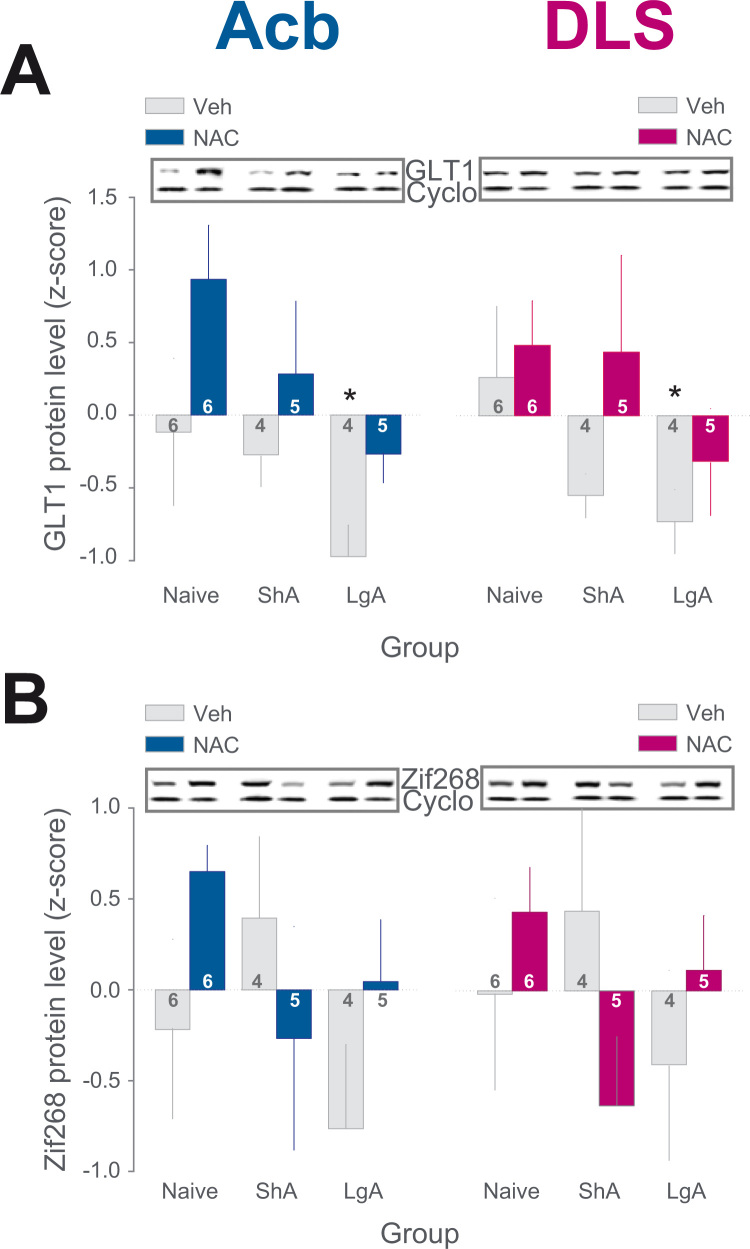
*N*-acetylcysteine (NAC) rescues cocaine-induced decreases in glutamate type 1 transporter (GLT1) protein levels and increases those of Zif268 in both the ventral striatum and dorsolateral striatum (DLS) of long access group (LgA) rats. NAC rescued the access-dependent decrease in GLT1 protein levels following cocaine self-administration both in the nucleus accumbens core (AcbC) and DLS (main effect of access: *F*_2,24_ = 4.73, *p* < .02, partial η^2^ = .28, and treatment: *F*_1,24_ = 6.27, *p* < .02, partial η^2^ = .2, but no structure × access or access × treatment × structure interactions: *F*s < 1) **(A)**. NAC also counteracted the effect of either short or extended access to cocaine self-administration on the protein levels of the plasticity marker Zif268 in the AcbC and DLS (treatment × access interaction: *F*_2,24_ = 4.26, *p* < .03, partial η^2^ = .26) **(B)**. Inserts show illustrative Western blots from the naive, short access group (ShA), and LgA groups for the protein of interest GLT1 and Zif268, as well as the housekeeping gene protein cyclophylin (Cyclo) obtained from micro-punches of the AcbC or DLS of individual rats. *Different from vehicle-treated drug naïve control rats, *p* < .05. Veh, vehicle.

The apparent restoration of control in LgA rats promoted by NAC after punishment was accompanied by a rescue of the access-dependent decrease in GLT1 protein levels observed progressively to develop after short- and long-term cocaine exposure not only in the AcbC but also in the DLS (Figure 4A).

Thus, in LgA rats, in which GLT1 protein levels were significantly reduced in both the AcbC and DLS, NAC treatment increased GLT1 protein levels back to the level of drug-naïve vehicle control subjects.

This effect of chronic NAC treatment on GLT1 protein levels across the striatum was paralleled by a NAC-induced increase in Zif268 protein levels in both the AcbC and DLS of LgA rats, as compared with Veh-LgA rats, a finding opposite to the observed decrease in Zif268 following NAC treatment in ShA rats compared with Veh-ShA rats (Figure 4B). The level of both GLT1 and Zif268 proteins in the AcbC and DLS were not correlated with each other or with cocaine intake and instrumental performance overall (total intake or total amount of active lever presses from day 1) or during the three punished and the five subsequent baseline sessions.

## Discussion

This study investigated the effects of chronic NAC treatment on rats given extended access to cocaine, which resulted in their showing hallmark features of cocaine addiction, namely loss of control over drug intake, increased motivation for the drug, and maintained drug use despite adverse consequences (18). Chronic treatment with NAC had no measurable effect on the progression of loss of control in rats that had already developed escalated cocaine SA or on the motivation for the drug measured under a progressive ratio schedule of reinforcement. This observation is in contrast with the previous demonstration that chronic NAC treatment prevents the escalation of cocaine self-administration (13). This discrepancy may stem from several differences between the two studies including the training history of the animals, the unit dose of cocaine, and the time point at which NAC treatment was initiated. Thus, in the present study, rats acquired instrumental responding for cocaine infusions (.8 mg/kg in 100 µL) under short access, FR1 schedule of reinforcement for 7 days before being exposed to 6-hour extended access or maintained under short access conditions. In the previous study, rats received unit infusions of .5 mg/kg in 200 µL, a unit dose and delivery rate both shown to greatly influence various behavioral features of cocaine self-administration, including motivation (40). More importantly, whereas the rats of the previous study received daily NAC treatment from the first of the 12 days of differential access to cocaine, i.e., from the first session of extended access for the LgA group, in this study rats were allowed 19 extended access sessions to develop a robust escalation of cocaine SA before receiving NAC treatment. In this study, we were more interested in the influence of NAC on the maintenance of escalated cocaine SA in rats suggested to resemble addicted individuals and not in the development of escalation, which represents a prodromal stage of addiction and hence may not be a suitable target for therapeutic intervention. Thus, while NAC treatment interacts with the neurobiological mechanisms involved in the development of escalation of cocaine SA (13), it does not influence its maintenance. This observation is in line with human studies that have revealed that NAC treatment does not decrease ongoing cocaine use (4).

However, chronic NAC treatment in rats showing escalated cocaine intake dramatically reduced compulsivity by enabling LgA rats to withhold responding in the face of punishment, thereby achieving abstinence more readily. Not only did NAC-treated LgA rats display a faster decrease in cocaine SA than Veh-treated LgA rats in the presence of contingent punishment, they also never fully recovered their prepunishment level of cocaine intake over the course of the five subsequent baseline sessions. This decrease in intake following punishment when the drug was again made available at no cost suggests that NAC not only facilitated the establishment of abstinence in the face of adverse consequences but also promoted long-term reduction in intake. This was paralleled by a marked increase in the latency to complete the first FR5 sequence leading to cocaine infusions over the five postpunishment sessions, suggesting that NAC-treated LgA rats displayed an increased propensity to inhibit the drug-seeking instrumental sequence for a longer period of time postpunishment than Veh-treated LgA rats.

This effect of NAC on the sensitivity to the aversive consequences of cocaine SA observed only in LgA rats is attributable neither to the differential access to the drug nor to a direct pharmacologic effect of NAC on pain thresholds. Indeed, the emergence of compulsive cocaine SA has recently been shown not to be associated with an increase in pain threshold (41), an effect consistent with evidence that enhanced resistance to conditioned suppression after an extended cocaine SA history is not related to individual sensitivity to the aversive properties of the shock (42). Additionally, NAC diminishes the sensitivity to painful stimuli (43, 44), i.e., increases pain thresholds so that if the effect of NAC was dependent on its effect on pain mechanisms, a decrease in, rather than a potentiation of, the suppression of instrumental responding during punishment under NAC treatment would be expected, and this would be generalized both to ShA and LgA rats.

The promotion of restored control induced by NAC in LgA rats was independent of any alteration in the motivation for cocaine as measured by the breakpoint under a progressive ratio schedule of reinforcement. Moreover, the breakpoint and the suppression of cocaine intake following punishment were not correlated. These data are in agreement with the previous demonstration that NAC reduces cue-controlled cocaine seeking without altering cocaine intake under a second-order schedule of reinforcement (6), suggesting that NAC has no effect on the reinforcing properties of cocaine despite the increased propensity to lever press under a progressive ratio schedule of reinforcement shown by LgA rats as compared with ShA rats.

Thus, NAC may enhance the long-lasting effects of being exposed to the punishment of responding for cocaine on the subsequent propensity to resume drug use but only in individuals that have lost control over intake. Indeed, LgA rats displayed the key behavioral characteristics of cocaine addiction: they developed a daily increase in their intake, so-called escalation of cocaine SA (16, 19); they displayed an apparent increased motivation for cocaine as compared with ShA rats consistent with previous studies (45, 46, 47); and they were less sensitive to punishment than ShA rats during the first punishment session, thereby revealing the compulsive nature of their cocaine SA (18).

Protracted exposure to extended access to cocaine, in conditions similar to those in the present experiment, triggered an almost complete resistance to probabilistic punishment in a subset of a population of rats tested in a seeking-taking chained schedule of reinforcement (48). The greater sensitivity to punishment in the present experiment may be attributed to the nature of the punishment applied [see (18, 21) for more details]. In the procedure used here, the animal does not risk punishment to gain access to the drug, as is the case in probabilistic punishment paradigms, but instead it must endure at least two shocks before every single infusion, making the latter punishment schedule more aversive than the former. Nevertheless, as previously described (48, 49), extended access to the drug facilitated the emergence of compulsive cocaine self-administration. In drug-addicted humans, voluntary abstinence usually arises only after repeated exposure to adverse consequences of drug use (50). Even though initially resisting punishment more than ShA rats, LgA rats developed complete abstinence after three repeated punishment sessions. This withholding of responses, or abstinence, in the presence of adverse consequences has been suggested to have face and ecological validity (18, 51).

At the neurobiological level, cocaine exposure resulted in an access-dependent decrease in GLT1 both in the AcbC, as previously described (14, 52), and in the DLS. The access-dependent decrease in GLT1 was quantitatively similar between the AcbC and the DLS and was reversed in both striatal territories by chronic NAC treatment. This influence of NAC on GLT1 protein levels had already been shown to occur in the AcbC (2, 11, 14, 53) but not in the DLS. In this study, protein levels were quantified in rats having been given access to cocaine under baseline conditions after having voluntarily withheld self-administering responses in the face of punishment. Additionally, neither Zif268 nor GLT1 protein levels correlated with cocaine intake or instrumental performance overall or during the punished or subsequent baseline sessions. Thus, while this study identifies the DLS as an additional site at which NAC regulates glutamate homeostasis and cellular plasticity mechanisms while contributing to restoration of control over cocaine intake in addicted individuals, it does not allow any inference about a causal relationship between the molecular and behavioral readouts. Future research is warranted to identify the cascade of cellular and molecular events, such as alteration of the cysteine-glutamate exchanger (54) and metabotropic glutamate receptors levels (55) that lead chronic NAC treatment eventually to facilitate self-initiated abstinence and subsequent restoration of control over intake.

Nevertheless, the influence of cocaine exposure on the progressive decrease in GLT1 protein levels in the DLS and its reversal by NAC is in line with the evidence that NAC diminishes habitual control over behavior (56), which is dependent upon the DLS (57), and our previous demonstration that NAC dose-dependently decreases habitual cocaine seeking in rats trained extensively to seek cocaine under the control of drug-associated conditioned stimuli (6). These conditions are known to trigger a shift in the neural locus of control over seeking responses from the ventral striatum to the DLS involving both glutamatergic and dopaminergic mechanisms (6, 27, 58). The devolution of control over cocaine seeking to the DLS is also associated with the development of compulsive cocaine seeking (59), which is facilitated in rats with a history of escalated cocaine self-administration (48).

This ventral to dorsal striatal shift in the locus of control over compulsive drug seeking has also been shown in drug-addicted individuals (25) who also display an increased functional connectivity between the AcbC and DLS (26), suggested to be triggered by drug exposure (60). This view is compatible with the influence of NAC treatment on cocaine-induced alterations in the protein levels of the cellular plasticity marker Zif268 (35) observed between short and extended cocaine exposures that were independent of GLT1 protein levels, as revealed by a lack of correlation between the two markers in both the AcbC and DLS. Thus, while NAC reversed the increase in Zif268 observed in the AcbC and the DLS following short access to cocaine, it abolished the decrease in Zif268 observed in both striatal territories following extended access. These data suggest that the neurobiological locus underlying the behavioral effects of NAC in addicted rats lies both in the AcbC and DLS in which cellular Zif268-dependent plasticity mechanisms, involved in instrumental learning and the long-term cellular effects of cocaine (31, 32, 33, 34, 35, 36), are differentially regulated by short or extended access to cocaine. Thus, short access to cocaine results in similar increases in Zif268-associated cellular plasticity mechanisms in the AcbC and DLS that may reflect drug-induced striatal plasticity mechanisms subserving the early effects of cocaine exposure. However, under extended access conditions, when rats lose control over cocaine self-administration, becoming compulsive, the striatal system may become resilient to cocaine-induced cellular plasticity as previously described (61), so-called anaplasticity, which could be related with the persistence of an aberrant functional coupling between the ventral striatum and the DLS, also seen in drug-addicted humans (25), that is disrupted by NAC treatment. As a consequence, animals may re-establish control over their drug-seeking habits, illustrated in this study by the increased time NAC-LgA rats take to perform their first cocaine-seeking sequence in the sessions that follow their self-imposed abstinence.

If the reorganization of glutamatergic function brought about by NAC treatment in both the ventral striatum and DSL provides a mechanism by which control over drug intake can be regained, then other defining features of addiction may also be attenuated.

## References

[1] Kalivas P.W. (2009). The glutamate homeostasis hypothesis of addiction. Nat Rev Neurosci.

[2] Brown R.M., Kupchik Y.M., Kalivas P.W. (2013). The story of glutamate in drug addiction and of N-acetylcysteine as a potential pharmacotherapy. JAMA Psychiatry.

[3] Baker D., McFarland K., Lake R., Shen H., Toda S., Kalivas P. (2003). N-acetyl cysteine-induced blockade of cocaine-induced reinstatement. Ann N Y Acad Sci.

[4] LaRowe S., Kalivas P., Nicholas J., Randall P., Mardikian P., Malcolm R. (2013). A double-blind placebo-controlled trial of N-acetylcysteine in the treatment of cocaine dependence. Am J Addict.

[5] Murray J, Lacoste J, Belin D. (2012): N-Acetylcysteine as a treatment for addiction. In: Belin D, editor. Addictions: From Pathophysiology to Treatment. Rijeka, Croatia: InTech, 335–380.

[6] Murray J., Everitt B., Belin D. (2012). N-Acetylcysteine reduces early- and late-stage cocaine seeking without affecting cocaine taking in rats. Addict Biol.

[7] Schmaal L., Veltman D., Nederveen A., van den Brink W., Goudriaan A. (2012). N-acetylcysteine normalizes glutamate levels in cocaine-dependent patients: A randomized crossover magnetic resonance spectroscopy study. Neuropsychopharmacology.

[8] Martinez D., Slifstein M., Nabulsi N., Grassetti A., Urban N.B., Perez A. (2014). Imaging glutamate homeostasis in cocaine addiction with the metabotropic glutamate receptor 5 positron emission tomography radiotracer [(11)C]ABP688 and magnetic resonance spectroscopy. Biol Psychiatry.

[9] Yang S., Salmeron B.J., Ross T.J., Xi Z.X., Stein E.A., Yang Y. (2009). Lower glutamate levels in rostral anterior cingulate of chronic cocaine users - A (1)H-MRS study using TE-averaged PRESS at 3 T with an optimized quantification strategy. Psychiatry Res.

[10] LaRowe S., Mardikian P., Malcolm R., Myrick H., Kalivas P., McFarland K. (2006). Safety and tolerability of N-acetylcysteine in cocaine-dependent individuals. Am J Addict.

[11] Kupchik Y., Moussawi K., Tang X., Wang X., Kalivas B., Kolokithas R. (2012). The effect of N-acetylcysteine in the nucleus accumbens on neurotransmission and relapse to cocaine. Biol Psychiatry.

[12] Brown R., Kupchik Y., Kalivas P. (2013). The story of glutamate in drug addiction and of N-acetylcysteine as a potential pharmacotherapy. JAMA Psychiatry.

[13] Madayag A., Lobner D., Kau K., Mantsch J., Abdulhameed O., Hearing M. (2007). Repeated N-acetylcysteine administration alters plasticity-dependent effects of cocaine. J Neurosci.

[14] Fischer K.D., Houston A.C., Rebec G.V. (2013). Role of the major glutamate transporter GLT1 in nucleus accumbens core versus shell in cue-induced cocaine-seeking behavior. J Neurosci.

[15] Peck J.A., Ranaldi R. (2014). Drug abstinence: Exploring animal models and behavioral treatment strategies. Psychopharmacology (Berl).

[16] Dilleen R., Pelloux Y., Mar A., Molander A., Robbins T., Everitt B. (2012). High anxiety is a predisposing endophenotype for loss of control over cocaine, but not heroin, self-administration in rats. Psychopharmacology (Berl).

[17] Paterson N.E., Markou A. (2003). Increased motivation for self-administered cocaine after escalated cocaine intake. Neuroreport.

[18] Belin-Rauscent A., Fouyssac M., Bonci A., Belin D. (2016). How preclinical models evolved to resemble the diagnostic criteria of drug addiction. Biol Psychiatry.

[19] Ahmed S.H., Koob G. (1998). Transition from moderate to excessive drug intake: Change in hedonic set point. Science.

[20] Belin D., Mar A.C., Dalley J.W., Robbins T.W., Everitt B.J. (2008). High impulsivity predicts the switch to compulsive cocaine-taking. Science.

[21] Belin D., Deroche-Gamonet V. (2012). Responses to novelty and vulnerability to cocaine addiction: Contribution of a multi-symptomatic animal model. Cold Spring Harb Perspect Med.

[22] Scofield M.D., Boger H.A., Smith R.J., Li H., Haydon P.G., Kalivas P.W. (2015). Gq-DREADD selectively initiates glial glutamate release and inhibits cue-induced cocaine seeking. Biol Psychiatry.

[23] Moussawi K., Pacchioni A., Moran M., Olive M., Gass J., Lavin A., Kalivas P.W. (2009). N-Acetylcysteine reverses cocaine-induced metaplasticity. Nat Neurosci.

[24] Belin D., Belin-Rauscent A., Murray J.E., Everitt B.J. (2013). Addiction: Failure of control over maladaptive incentive habits. Curr Opin Neurobiol.

[25] Vollstadt-Klein S., Wichert S., Rabinstein J., Buhler M., Klein O., Ende G. (2010). Initial, habitual and compulsive alcohol use is characterized by a shift of cue processing from ventral to dorsal striatum. Addiction.

[26] Xie C., Shao Y., Ma L., Zhai T., Ye E., Fu L. (2014). Imbalanced functional link between valuation networks in abstinent heroin-dependent subjects. Mol Psychiatry.

[27] Belin D., Everitt B.J. (2008). Cocaine seeking habits depend upon dopamine-dependent serial connectivity linking the ventral with the dorsal striatum. Neuron.

[28] Hollander J., Im H., Amelio A., Kocerha J., Bali P., Lu Q. (2010). Striatal microRNA controls cocaine intake through CREB signalling. Nature.

[29] Zapata A., Minney V., Shippenberg T. (2010). Shift from goal-directed to habitual cocaine seeking after prolonged experience in rats. J Neurosci.

[30] Willuhn I., Burgeno L., Everitt B., Phillips P. (2012). Hierarchical recruitment of phasic dopamine signaling in the striatum during the progression of cocaine use. Proc Natl Acad Sci U S A.

[31] Maroteaux M., Valjent E., Longueville S., Topilko P., Girault J.A., Herve D. (2014). Role of the plasticity-associated transcription factor zif268 in the early phase of instrumental learning. PLoS One.

[32] Veyrac A., Besnard A., Caboche J., Davis S., Laroche S. (2014). The transcription factor Zif268/Egr1, brain plasticity, and memory. Prog Mol Biol Transl Sci.

[33] Unal C.T., Beverley J.A., Willuhn I., Steiner H. (2009). Long-lasting dysregulation of gene expression in corticostriatal circuits after repeated cocaine treatment in adult rats: Effects on zif 268 and homer 1a. Eur J Neurosci.

[34] Knapska E., Kaczmarek L. (2004). A gene for neuronal plasticity in the mammalian brain: Zif268/Egr-1/NGFI-A/Krox-24/TIS8/ZENK?. Prog Neurobiol.

[35] Valjent E., Aubier B., Corbille A.G., Brami-Cherrier K., Caboche J., Topilko P. (2006). Plasticity-associated gene Krox24/Zif268 is required for long-lasting behavioral effects of cocaine. J Neurosci.

[36] Moratalla R., Robertson H., Graybiel A. (1992). Dynamic regulation of NGFI-A (zif268, egr1) gene expression in the striatum. J Neurosci.

[37] Murray J.E., Dilleen R., Pelloux Y., Economidou D., Dalley J.W., Belin D., Everitt B.J. (2014). Increased impulsivity retards the transition to dorsolateral striatal dopamine control of cocaine seeking. Biol Psychiatry.

[38] Levine T.R., Hullett C.R. (2002). Eta squared, partial eta squared, and misreporting of effect size in communication research. Hum Commun Res.

[39] Belin D., Berson N., Balado E., Piazza P.V., Deroche-Gamonet V. (2011). High-novelty-preference rats are predisposed to compulsive cocaine self-administration. Neuropsychopharmacology.

[40] Allain F., Minogianis E.A., Roberts D.C., Samaha A.N. (2015). How fast and how often: The pharmacokinetics of drug use are decisive in addiction. Neurosci Biobehav Rev.

[41] Vanhille N, Ducret E, Puaud M, Ansquer S, Fouyssac M, Houeto J, *et al*. (2013): C.20 - Differential effect of environmental enrichment on the acquisition of drug use and the transition to addiction. Presented at the 15th Biennal Meeting of the European Behavioural Pharmacology Society, September 6-9, La Rochelle, France.

[42] Vanderschuren L.J., Everitt B.J. (2004). Drug seeking becomes compulsive after prolonged cocaine self-administration. Science.

[43] Chu C., Levine E., Gear R.W., Bogen O., Levine J.D. (2011). Mitochondrial dependence of nerve growth factor-induced mechanical hyperalgesia. Pain.

[44] Naik A.K., Tandan S.K., Dudhgaonkar S.P., Jadhav S.H., Kataria M., Prakash V.R., Kumar D. (2006). Role of oxidative stress in pathophysiology of peripheral neuropathy and modulation by N-acetyl-L-cysteine in rats. Eur J Pain.

[45] Hao Y., Martin-Fardon R., Weiss F. (2010). Behavioral and functional evidence of metabotropic glutamate receptor 2/3 and metabotropic glutamate receptor 5 dysregulation in cocaine-escalated rats: Factor in the transition to dependence. Biol Psychiatry.

[46] Specio S.E., Wee S., O’Dell L.E., Boutrel B., Zorrilla E.P., Koob G.F. (2008). CRF(1) receptor antagonists attenuate escalated cocaine self-administration in rats. Psychopharmacology (Berl).

[47] Orio L., Edwards S., George O., Parsons L.H., Koob G.F. (2009). A role for the endocannabinoid system in the increased motivation for cocaine in extended-access conditions. J Neurosci.

[48] Pelloux Y., Everitt B.J., Dickinson A. (2007). Compulsive drug seeking by rats under punishment: Effects of drug taking history. Psychopharmacology (Berl).

[49] Jonkman S., Pelloux Y., Everitt B. (2012). Drug intake is sufficient, but conditioning is not necessary for the emergence of compulsive cocaine seeking after extended self-administration. Neuropsychopharmacology.

[50] Waldorf D., Reinarman C., Murphy S. (1991). Cocaine Changes: The Experience of Using and Quitting.

[51] Economidou D., Pelloux Y., Robbins T., Dalley J., Everitt B. (2009). High impulsivity predicts relapse to cocaine-seeking after punishment-induced abstinence. Biol Psychiatry.

[52] Fischer-Smith K.D., Houston A.C., Rebec G.V. (2012). Differential effects of cocaine access and withdrawal on glutamate type 1 transporter expression in rat nucleus accumbens core and shell. Neuroscience.

[53] Scofield M., Kalivas P. (2014). Astrocytic dysfunction and addiction: Consequences of impaired glutamate homeostasis. Neuroscientist.

[54] Moran M., McFarland K., Melendez R., Kalivas P., Seamans J. (2005). Cystine/glutamate exchange regulates metabotropic glutamate receptor presynaptic inhibition of excitatory transmission and vulnerability to cocaine seeking. J Neurosci.

[55] Uys J., LaLumiere R. (2008). Glutamate: The new frontier in pharmacotherapy for cocaine addiction. CNS Neurol Disord Drug Targets.

[56] Corbit L.H., Chieng B.C., Balleine B.W. (2014). Effects of repeated cocaine exposure on habit learning and reversal by N-acetylcysteine. Neuropsychopharmacology.

[57] Yin H.H., Knowlton B.J., Balleine B.W. (2004). Lesions of dorsolateral striatum preserve outcome expectancy but disrupt habit formation in instrumental learning. Eur J Neurosci.

[58] Vanderschuren L., Di Ciano P., Everitt B. (2005). Involvement of the dorsal striatum in cue-controlled cocaine seeking. J Neurosci.

[59] Jonkman S., Pelloux Y., Everitt B. (2012). Differential roles of the dorsolateral and midlateral striatum in punished cocaine seeking. J Neurosci.

[60] Takahashi Y., Schoenbaum G., Niv Y. (2008). Silencing the critics: Understanding the effects of cocaine sensitization on dorsolateral and ventral striatum in the context of an actor/critic model. Front Neurosci.

[61] Kasanetz F., Deroche-Gamonet V., Berson N., Balado E., Lafourcade M., Manzoni O. (2010). Transition to addiction is associated with a persistent impairment in synaptic plasticity. Science.

