# Supplementary material for: N-acetylcysteine Facilitates Self-Imposed Abstinence After Escalation of Cocaine Intake
Source: Biol Psychiatry. 2016 Aug 1;80(3):226–34. doi: 10.1016/j.biopsych.2015.09.019 (PMC4954758; doi:10.1016/j.biopsych.2015.09.019)
Supplement: Supplementary file 1 — Supplementary Material [file mmc1.pdf]

N-acetylcysteine Facilitates Self-Imposed Abstinence After Escalation of Cocaine Intake

Supplemental Information

Supplemental Methods and Materials

Animals

Rats were housed two per cage throughout the experiment in a reversed 12 hour light/dark cycle (light off at 7 am). Rats had free access to water and were fed 20 g/rat/day of standard chow pellets within one hour of each training session. Rats were weighed prior to the self-administration sessions.

Solutions

Cocaine hydrochloride (Coopération Pharmaceutique Française) was dissolved in sterile 0.9% NaCl (CEVA, France).

N-acetylcysteine (NAC, Sigma-Aldrich, France) was dissolved in sterile distilled water (60 mg/kg), a dose that has been shown to decrease AcbC and dorsolateral striatum (DLS)-dependent cocaine seeking behavior (1, 2) and was administered daily intraperitoneally (IP, 1 ml/kg) 3 hours prior to testing sessions (2) as this interval has been shown to result in increased extrasynaptic brain glutamate levels at the time of testing (see 1).

Surgery

Rats were anaesthetized with an IP injection of ketamine and xylazine (90 and 6.7 mg/kg respectively) and implanted with a catheter inserted in the right atrium through the jugular vein. Following the surgery, rats were treated for one week with daily subcutaneous injection of an antibiotic (0.2 mL of Baytril, Bayer) and the catheter flushed with 0.1 ml of heparinized saline (50 U/ml in 0.9% sterile saline; Sanofi-Aventis).

Apparatus

The twelve operant chambers (31.8 cm long x 25.4 cm width x 34.3 cm high) in plexiglas with a metal grid floor were housed in ventilated, sound-attenuating cubicles (Med Associates Inc., Sandown Scientific Ltd.). Two retractable levers were surmounted with a cue light and a third cue light was located between the two levers, on top of the wall. A white house light, situated at the opposite wall of the levers, allowed complete illumination of the chamber. Implanted catheters were connected to a syringe driven by an infusion pump

(Semat Technical, Herts, UK) via Tygon tubing. The infusion speed was 20 µL/s.

Procedures

The reliability of the behavioral effects of NAC was assessed by performing the same experiment twice, on two independent cohorts of 24 rats, each experiment independently revealing the same behavioral effects of NAC treatment. Upon evidence that the baseline performance was similar between the two experiments, they were pooled and presented here as a single data set.

Cocaine Self-Administration. A week following catheter implantation the 48 rats used in the behavioral experiment were given seven daily sessions of cocaine SA under a fixed ratio 1 (FR1) schedule of reinforcement. Sessions started with the illumination of the chamber and the presentation of the two levers. Presses on the active lever turned on the white cue located above it for 5 s (conditioned stimulus, CS) and was reinforced with an infusion of cocaine (0.8 mg/kg/infusion). Drug infusions were followed by a 40 s time-out period during which both levers were retracted. Presses on the inactive lever were without scheduled consequences. They were then randomly assigned to either short (ShA, 1 h) or long access (LgA, 6 h) daily sessions of cocaine SA. From session 19 of differential access (a time at which a robust escalation was observed in LgA rats) to the end of the experiment, ShA and LgA rats received daily IP injections (1 ml/kg) of either NAC (60 mg/kg) or vehicle (Veh) three hours before behavioral training as previously described (2). The response requirement was increased to 5 (FR5) from session 27 onward.

Motivation. A progressive ratio schedule of reinforcement (PR) was conducted following 5 sessions of FR5. Cocaine availability was signaled by the illumination of the chamber and the number of lever presses required to obtain one injection of cocaine was increased after each infusion according to the following progression (3, 4): 10, 20, 30, 45, 65, 85, 115, 145, 185, 225, 275, 325, 385, 445, 515, 585, 665, 745, 835, 925, 1025, 1125, 1235, 1345, 1465, 1585. The break point corresponded to the last ratio completed, i.e. the maximal number of responses that a rat performed to obtain one infusion of the same dose of cocaine (5). The session ceased after either 5 h or when a period of 1 h elapsed since the previously earned infusion.

Punishment. Following the PR test of motivation rats were given two baseline FR5 sessions prior to being exposed to three consecutive 1 h sessions during which cocaine seeking was punished by contingent electric foot-shocks. The chamber was illuminated by the house light and the first press on the active lever turned on the cue light located between the two levers, indicating the presence of the shock. After completion of a FR4, an electric foot shock (0.3 mA, 1 s) was delivered. When the rats performed a FR5, they received another foot shock

followed by a cocaine infusion associated with the CS. Then, the cue light signaling the shock was turned off and the sequence was reinitiated after a time-out period of 40 s. If, within a minute, the animals did not reach a FR4 or a FR5 the schedule was reinitiated (5).

Resumption of Cocaine Self-Administration. Rats were returned to FR5 baseline self-administration conditions.

All data were collected with PC-compatible software (MED-PC® IV, Med Associates Inc., Sandown Scientific Ltd.).

Western Blot

Brain Samples. Ninety minutes after the last behavioral session, rats from the second SA cohort were decapitated and their brains were harvested and frozen in -40°C isopentane (Sigma-Aldrich, France). Brains were also collected from drug naïve vehicle- (*n* = 6) or NAC-treated (*n* = 6) controls matching the cocaine-exposed rats for treatment, age and weight. Brains were stored at -80°C until processing. Bilateral punches of the nucleus accumbens core (6) and the anterior part of the DLS (2, 7) were taken from coronal slices (300 µm thick) cut at -18°C with a cryostat (Leica, France).

Protein Extraction and Assay. Punches were pooled and homogenized in lysis buffer (cOmplete Lysis-M, Roche) by repeated suction with a 20 µL pipette before and after 45 min of freezing at -20°C. After a centrifugation (15000 *g*) for 15 min at 4°C, supernatants were removed and the protein concentrations were assayed using the Qubit kit (Invitrogen, France).

Electrophoresis. Proteins were diluted at a final concentration of 1 g/L in LDS NuPAGE buffer (Invitrogen-Life Technologies, France) added with DTT (0.05 M) and water. The samples were heated 5 min at 70°C and 20 µg of protein were loaded onto 4-20% NuPAGE Tris-Glycine gel (Invitrogen-Life Technologies, France). One well contained the ladder for determining molecular weights (EZ-Run prestained recombinant protein, Fisher Bioreagents).

Incubations and Revelations. Following the transfer of proteins on nitrocellulose membranes (Invitrogen-Life Technologies, France) with an iBlot device (Invitrogen) for 7 min at 20V, the membranes were washed in Tris-buffer saline (Euromedex) containing Tween 20 0.05% (TBST) for 10 min and incubated for 2 hours in blocking solution (BSA: 5% in TBST) before incubation with the primary antibodies overnight at 4°C. The following primary antibodies were used: anti-Egr-1 (1/750, Santa Cruz, sc-110) and anti-GLT1 (1/6000, Millipore, #ABN102). After washing the membranes in TBST, blots were incubated in an anti-rabbit secondary antibody (1/1000, Cell Signaling, 7074S) for 1 h at room temperature.

Finally, blots were revealed using Lumintata Western HRP Substrates (Millipore) and the signal was acquired with a G:BOX (Syngene) coupled with the GeneSnap software. The optical density of each band was quantified using the GeneTools software (Syngene).

Each western blot was run in triplicate. The optical density (OD) of each band was normalized by the amount of cyclophiline A present in each well, to correct variations in the quantity of proteins loaded, and the resulting ratio was used to assess protein concentrations. OD × area calculation was performed to derive an amount of protein. The measurements having been replicated on three different membranes, upon confirmation of the consistency of the signal across independent experiments; the protein amount computed from each replicate was averaged and used for statistical analysis.

Data and Statistical Analyses

Data are expressed as means ± SEM and were analyzed using the StatSoft Statistica 9 statistics package.

Of the 48 rats initially trained to self-administer cocaine on a FR1, twelve rats were removed from the final analyses due to catheter failure over the course of the 49 days of training.

For analysis of the behavioral data, repeated-measures analyses of variance (ANOVAs) were used with access to cocaine (ShA vs LgA) and treatment (NAC vs Veh) as between-subject factors and sessions of self-administration or pre / post treatment blocks as within-subject factors.

Western blot data were subjected to factorial ANOVAs with treatment (Veh vs NAC) and cocaine exposure (Naïve, ShA, LgA) as between-subject factors and, when stated, structures (AcbC and DLS) as within-subject factor.

Potential dimensional relationships between cellular markers (z-scores of Zif268 and GLT1 protein levels) and behavior (cocaine intake or active lever presses) were analyzed across access and treatment groups using the Spearman correlation.

Normal distributions of the populations were tested with a Kolmogorov-Smirnov test before performing any correlations. If the population followed a bimodal distribution, the test of normality and correlation was performed separately on the two subgroups. Homogeneity of variance was assessed with the Levene test and sphericity was assessed with the Mauchly sphericity test. When the assumption of sphericity or homogeneity of variance was violated F and p values were corrected with the Huynh & Feldt method or log-transformed, respectively.

The suppression score was calculated as one minus the ratio between the average intake of the two days following the punished sessions and the intake during the two sessions preceding the punished sessions. Cluster analysis was conducted on the

suppression score using the k-means clustering method with 10 iterations. We made the assumption that the animals could be distributed into four different clusters: one with a high suppression score, a second with a moderate suppression score (under 0.5), a third group with a score of 0 (displaying no suppression of the instrumental response) and a fourth group with a negative score (corresponding to a potentiation of the instrumental response following the punishment sessions).

To assess the maintenance of escalation of cocaine SA we used the slope of drug intake (mg/kg) over the different periods of long access (pre- and post-treatment). The escalation slope is a reliable and reproducible measure of escalation (8). A positive slope indicates an overall increase in intake over time, while a negative slope indicates a decrease in intake over time.

For the analysis of western blot data, the individual values were transformed in z-scores (Z-score = [average protein of interest level for rat_n - average protein of interest level for all rats]/standard deviation) as previously described for behavioral variables (3, 9-11) regardless the drug access or treatment conditions. This provides for each protein of interest a uniformity in the dimensionality of protein levels between the Acb and the DLS datasets, thereby offering an opportunity to directly compare the two structures independently of potential differences in expression level that may bias the general statistical design.

Supplemental Results

Acquisition of Cocaine SA and Development of Escalation

Rats acquired cocaine SA under FR1 within a week as revealed by the progressive increase in cocaine infusions over time [$F_{6, 210} = 8.08$, $p < .01$] (**Fig S1A**). The subsequent introduction of a differential access to cocaine resulted in a progressive and robust escalation of cocaine intake observed only in LgA rats [main effect of access: $F_{1, 34} = 641.23$, $p < .01$, and access x time interaction: $F_{18, 612} = 9.33$, $p < .01$] (**Fig S1B**). This increase in intake was apparent in LgA rats even during the first hour as compared to ShA rats [interaction time x access: $F_{18, 612} = 1.64$, $p < .05$] (**Fig S1C**).

Influence of Daily NAC Treatment on Cocaine Self-Administration

NAC had no effect on the escalation ratio of the LgA group [treatment x block $_{\text{before / during treatment}}$ interaction: $F_{1, 15} = 0.28$, and treatment x sessions x block $_{\text{before / during treatment}}$ interaction: $F_{7, 105} = 0.45$] (**Fig S2**).

Increasing the behavioral demand, by increasing the ratio upon which cocaine was

delivered from 1 to 5 (FR5), resulted in a proportional increase in active lever presses in all rats [main effect of ratio: $F_{1, 34}$ = 325.78, p < .01, access: $F_{1, 34}$ = 8.53, p < .01, lever: $F_{1, 34}$ = 60.56, p < .01 and ratio x access x lever interaction: $F_{1, 34}$ = 4.14, p < .05]. However, NAC had no effect on the propensity to engage in more effort to obtain the drug in both ShA and LgA rats [access x treatment x ratio x lever interaction: $F_{1, 32}$ < 1], the latter maintaining a level of intake much higher than the former [$F_{1, 32}$ = 36.80, p < .01].

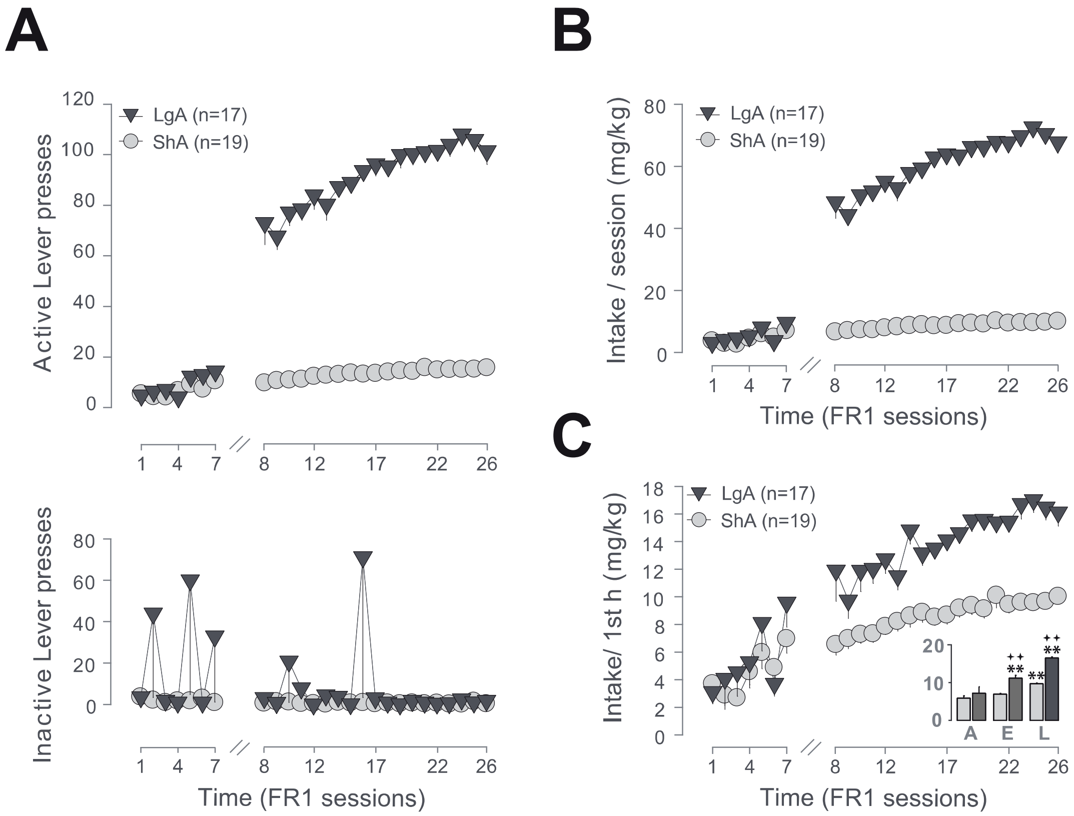

Figure S1. Rats with extended access to cocaine escalate cocaine self-administration display a robust escalation of drug intake over time. Compared with ShA rats, LgA rats displayed a robust increase in the number of active lever presses over the extended access daily sessions (**A**) which resulted in an increase of cocaine intake (**B**). Cocaine intake of LgA rats during the first hour of extended access also increased at a higher rate than the cocaine intake of the ShA rats (**C**), revealing an escalation of cocaine self-administration. Insert (**C**) represents the mean intake during the final three acquisition sessions and during the three first and last sessions of extended access (acquisition (A), early (E) and late (L) stage of differential access respectively, access x session interaction: $F_{2, 68}$ = 5.75, p < .05). ** p < .01 compared to acquisition, $^{++}$ p < .01 compared to ShA group. Breaks represent the introduction of differential access to cocaine.

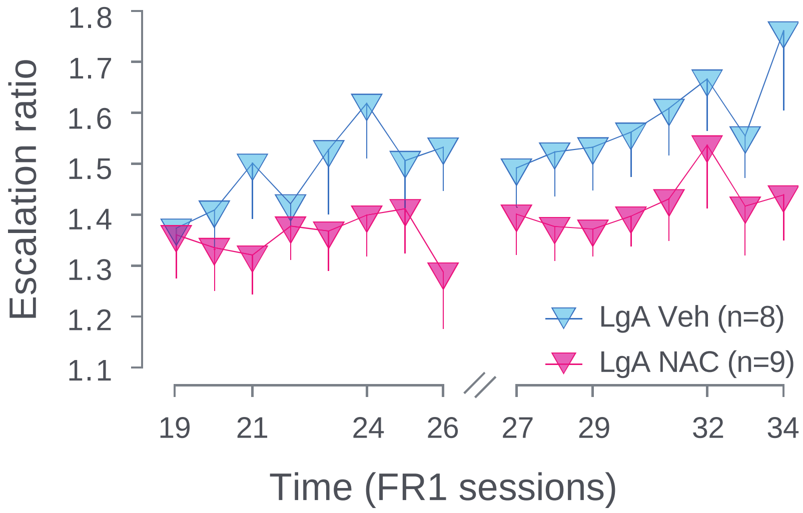

Figure S2. The escalation ratio was not influenced by NAC treatment. NAC treatment did not alter the escalation ratio of LgA rats calculated as the ratio of daily cocaine intake over the intake during the first session of extended access. Breaks represent the beginning of chronic IP injections of either NAC or vehicle.

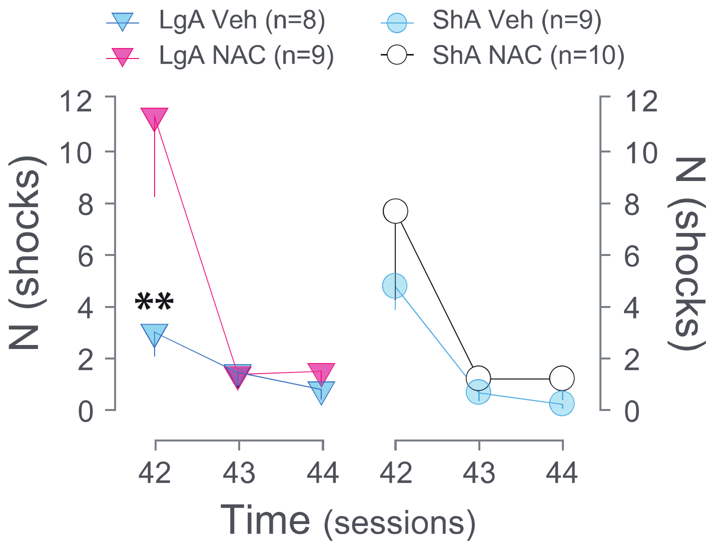

Figure S3. NAC decreased the number of punishments received by rats of the LgA group. NAC decreased the number of electric foot shocks received during the first punished session by LgA rats but was without effect on ShA rats. The number of foot shocks received during the two last sessions of punishment decreased for all groups regardless of treatment group [access x treatment x shock sessions interaction: $F_{2, 64} = 4.08$, $p < .05$]. ** $p < .01$.

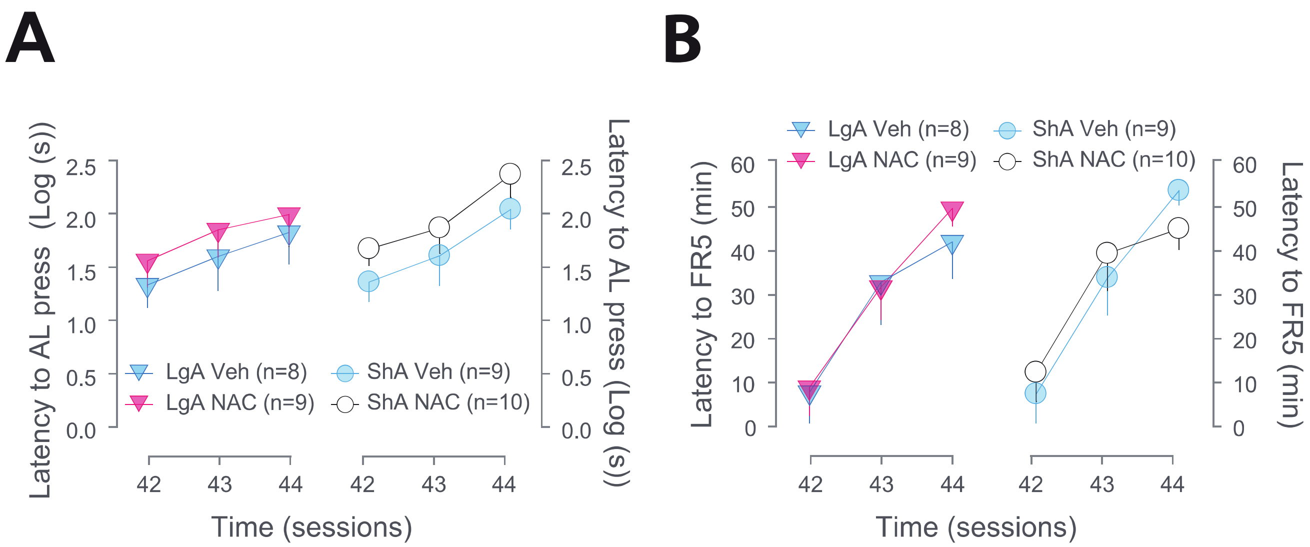

Figure S4. Punishment of cocaine SA increases the latency to initiate instrumental responses and the delay to obtain the first infusion. Both the latency to the first active lever press (**A**) and the time required to complete an instrumental sequence (FR5) (**B**) were increased in all groups across punishment sessions, regardless of treatment [main effect of time: $F_{2, 64} = 7.31$, $p < .01$ and $F_{2, 64} = 45.105$, $p < .001$; access x treatment x time interaction: $Fs_{2, 64} < 1$ for A and B respectively]

Supplemental References

1. Baker D, McFarland K, Lake R, Shen H, Toda S, Kalivas P (2003): N-acetyl cysteine-induced blockade of cocaine-induced reinstatement. *Ann N Y Acad Sci*. 1003:349-351.

2. Murray J, Everitt B, Belin D (2012): N-Acetylcysteine reduces early- and late-stage cocaine seeking without affecting cocaine taking in rats. *Addict Biol*. 17:437-440.

3. Belin D, Mar AC, Dalley JW, Robbins TW, Everitt BJ (2008): High impulsivity predicts the switch to compulsive cocaine-taking. *Science*. 320:1352-1355.

4. Deroche-Gamonet V, Belin D, Piazza PV (2004): Evidence for addiction-like behavior in the rat. *Science*. 305:1014-1017.

5. Belin D, Deroche-Gamonet V (2012): Responses to novelty and vulnerability to cocaine addiction: contribution of a multi-symptomatic animal model. *Cold Spring Harbor perspectives in medicine*. 2.

6. Fischer KD, Houston AC, Rebec GV (2013): Role of the major glutamate transporter GLT1 in nucleus accumbens core versus shell in cue-induced cocaine-seeking behavior. *J Neurosci*. 33:9319-9327.

7. Murray JE, Belin D, Everitt BJ (2012): Double dissociation of the dorsomedial and dorsolateral striatal control over the acquisition and performance of cocaine seeking. *Neuropsychopharmacology*. 37:2456-2466.

8. Dilleen R, Pelloux Y, Mar A, Molander A, Robbins T, Everitt B, *et al.* (2012): High anxiety is a predisposing endophenotype for loss of control over cocaine, but not heroin, self-administration in rats. *Psychopharmacology (Berl)*.89-89.

9. Belin D, Balado E, Piazza P, Deroche-Gamonet V (2009): Pattern of intake and drug craving predict the development of cocaine addiction-like behavior in rats. *Biol Psychiatry*. 65:863-868.

10. Belin D, Berson N, Balado E, Piazza PV, Deroche-Gamonet V (2011): High-novelty-preference rats are predisposed to compulsive cocaine self-administration. *Neuropsychopharmacology*. 36:569-579.

11. Namburi P, Beyeler A, Yorozu S, Calhoon GG, Halbert SA, Wichmann R, *et al.* (2015): A circuit mechanism for differentiating positive and negative associations. *Nature*. 520:675-678.
